# Genetic variants of major genes contributing to phosphate and calcium homeostasis and their association with serum parameters in pigs

**DOI:** 10.1007/s13353-018-0449-2

**Published:** 2018-06-22

**Authors:** Franziska Just, Henry Reyer, Eduard Muráni, Siriluck Ponsuksili, Michael Oster, Klaus Wimmers

**Affiliations:** 1 Institute of Genome Biology, Leibniz Institute for Farm Animal Biology, Wilhelm-Stahl-Allee 2, 18196 Dummerstorf, Germany; 2 Faculty of Agricultural and Environmental Sciences, University Rostock, 18059 Rostock, Germany

**Keywords:** Association analysis, Bone metabolism, Calcium, Phosphorus, Single nucleotide polymorphism

## Abstract

Calcium and phosphorus are irreplaceable components of life. Tracking the fate of calcium and phosphorus in organisms deserves high attention due to their relevance in bone metabolism and subsequently animal health. Indeed, bone serves as reservoir for calcium and phosphorus, whose formation and resorption follow specific molecular routes including hormones, receptors, and transcription factors. The objective of the study was to analyze the genetic variation of major components driving mineral utilization such as calcitonin receptor, calcium sensing receptor, fibroblast growth factor 23 (*FGF23*), parathyroid hormone receptor, osteopontin, stanniocalcin 1, RAF-type zinc finger domain containing 1 (*TRAFD1*), and vitamin D receptor. A German Landrace pig population (*n* = 360) was used to perform an association analysis between selected single nucleotide polymorphisms (SNP) and relevant serum parameters (calcium, phosphorus, calcium/phosphorus ratio, alkaline phosphatase). Analyzed SNPs in *FGF23* (rs710498025) and *TRAFD1* (rs345195312) were significantly (*p* ≤ 0.05) associated with the serum calcium/phosphorus ratio and serum phosphorus levels, respectively. This might represent a modulation of the homeostatic balance between calcium and phosphorus. Furthermore, *TRAFD1* is known to be involved in skeletal disorders which emphasize its link to phosphorus utilization and immune system. However, none of the analyzed genetic variants of these major regulators of phosphate and calcium homeostasis showed significant associations after correction for multiple testing (*q* value > 0.05). Thus, minor contributors as well as unknown and yet to be elucidated regulators of mineral homeostasis need to be characterized towards the implementation of improved phosphorus efficiency in pig breeding programs.

**Electronic supplementary material:**

The online version of this article (10.1007/s13353-018-0449-2) contains supplementary material, which is available to authorized users.

## Introduction

**Fig. 1:**
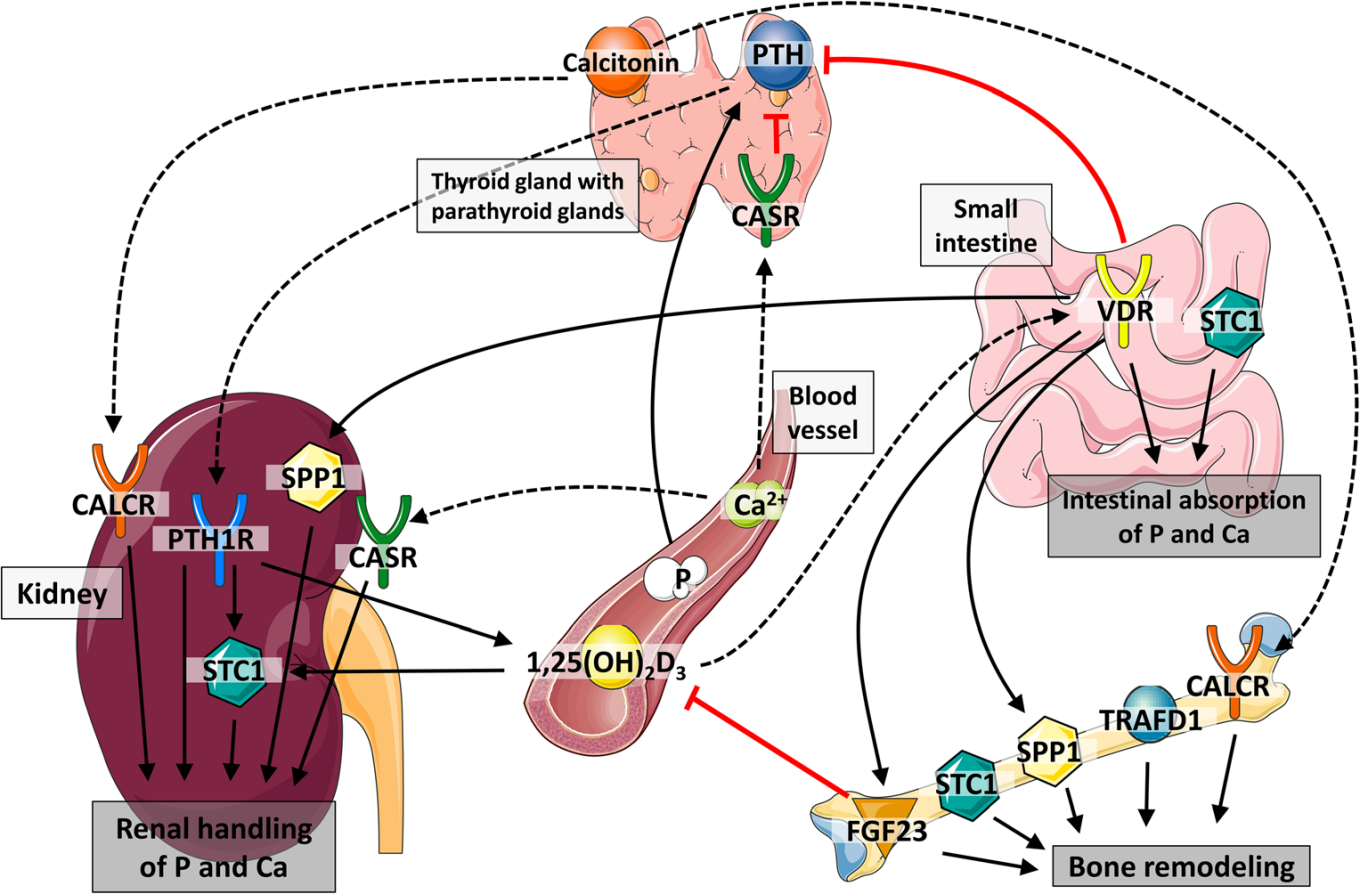
Interaction between candidate genes in phosphorus (P) and calcium (Ca) homeostasis. Dashed black lines indicate ligand-receptor interaction. Solid red lines indicate inhibition of hormones. Solid black lines indicate activation or involvement in process. See “Introduction” for further details. Abbreviations: CALCR calcitonin receptor, CASR calcium sensing receptor, FGF23 fibroblast growth factor 23, PTH1R parathyroid hormone receptor, SPP1 osteopontin, STC1 stanniocalcin 1, TRAFD1 TRAF-type zinc finger domain containing 1, VDR vitamin D receptor, 1,25(OH)_2_D_3_ calcitriol, P serum inorganic phosphate, Ca^2+^ serum calcium. Servier Medical Art (http://www.servier.com) was partly used to design the image

Welfare and health status of monogastric farm animals are closely related to bone and mineral metabolism. Indeed, skeletal disorders such as rickets and osteochondrosis occur in both young piglets with rapidly growing bones and adult pigs, resulting in lameness and culling and thus in economic losses (Stern et al. 1995; Fukawa and Kusuhara 2001). Moreover, bone mineralization is correlated with body weight and body composition (Rothammer et al. 2017). The bone consists of the protective cortical and the metabolic active trabecular bone, being also the reservoir for calcium (Ca) and phosphorus (P). The P and Ca homeostasis follows sophisticated patterns (Sapir-Koren and Livshits 2011) and molecular routes (Oster et al. 2016; Just et al. 2018) and interconnects a number of tissues and organs (Fig. 1). Endogenous signals are transmitted to peripheral tissue sites via responsive hormones, receptors, and transcription factors such as the calcitonin receptor (*CALCR*), calcium sensing receptor (*CASR*), fibroblast growth factor 23 (*FGF23*), parathyroid hormone receptor (*PTH1R*), osteopontin (*SPP1*), stanniocalcin 1 (*STC1*), TRAF-type zinc finger domain containing 1 (*TRAFD1*), and vitamin D receptor (*VDR*).

In this context, some studies identified candidate genes affecting the mineral homeostasis and stating the susceptibility for structural soundness and leg weakness traits in pigs (Fan et al. 2009; Laenoi et al. 2012; Rangkasenee et al. 2013). Specifically, Rangkasenee et al. (2013) detected a novel SNP in *PTH1R*, which was associated with osteochondrosis lesions. The receptor itself mediates the actions of the parathyroid hormone (PTH), which has a key role in the P and Ca homeostasis. In fact, low serum Ca levels stimulate PTH synthesis and *PTH1R* actions, resulting in an enhanced renal P excretion (Bergwitz and Jüppner 2010). Via activated *PTH1R*, PTH stimulates the hydroxylation of calcidiol to calcitriol (active vitamin D form). The vitamin D system is primarily known to be essential for its role in mineral homeostasis and bone metabolism. The net effect of calcitriol is an increase in the intestinal and renal absorption and reabsorption of Ca and P (Bergwitz and Jüppner 2010). These effects are mediated by the *VDR*, regulating the expression of numerous genes via binding to vitamin D response elements (VDRE). Examples are the inhibition of *PTH* transcription and activation of *FGF23* and *SPP1* expression (Haussler et al. 2011). The hormone FGF23 is thought to be primarily secreted by osteocytes and osteoblasts and reduces circulating calcitriol and serum phosphate levels (Bergwitz and Jüppner 2010). Moreover, *FGF23* expression below or above a certain threshold will result in disturbed bone metabolism. This indicates an essential role for FGF23 in regulating the bone P flux due to bone remodeling processes (Martin et al. 2012). Osteopontin (encoded by *SPP1*) is involved in the regulation of bone cell adhesion, osteoclast function, and matrix mineralization. *SPP1* is also highly expressed in the kidney, and the majority of the protein is found in the urine, likely inhibiting calcium oxalate formation (Giachelli and Steitz 2000). Other regulators of Ca metabolism are encoded by *CASR* and *CALCR*. In the parathyroid gland, CASR recognizes extracellular Ca concentrations in order to regulating the PTH synthesis as mentioned above. In the kidney, the receptor responds to altered Ca concentrations with modulation of the urinary excretion of mineral ions (Hebert et al. 1997). The peptide hormone calcitonin is secreted by the thyroid and its physiological effects are mediated by its receptor CALCR. These effects include the inhibition of osteoclast-mediated bone resorption and the increase of renal Ca excretion (Pondel 2000). Fan et al. (2009) demonstrated an association of SNPs in *CASR* and *CALCR* with structural soundness and leg locomotion traits. Similar to CALCR, TRAFD1 mediates the resorbing and secretory activity of osteoclasts (Witwicka et al. 2015). Consequently, *TRAFD1* has been recently proposed as candidate gene for bone mineral density (Gu et al. 2003). Moreover, TRAFD1 is an important feedback regulator of the immune response (Mashima et al. 2005), thus representing a connection between P homeostasis and immune system. Also of interest is *STC1*, as the encoded glycoprotein hormone stanniocalcin regulates P as well as Ca homeostasis in intestine and kidney (Ishibashi and Imai 2002). *STC1* expression is upregulated by PTH and calcitriol in the kidney (Hung et al. 2012). Previous experiments by Madsen et al. (1998) showed a decreased Ca absorption, while P absorption was increased in swine intestine, which may enhance the deposition of hydroxyapatite into bone. This is consistent with the finding that *STC1* is expressed in osteoblasts and chondrocytes, but not in osteoclasts (Ishibashi and Imai 2002).

Measuring serum minerals such as P and Ca is a feasible way to assess the P utilization in pigs. Serum P levels reflect the amount of ingested P from the diet (McDowell 2003). Serum Ca levels are inversely related to serum P levels and are interlinked by the above described regulators. The resulting Ca/P ratio in serum represents a biological marker to characterize P utilization (Koch and Mahan 1986) and should be independent from body weight. The alkaline phosphatase (ALP) is a marker for bone mineralization and of diagnostic usage for different bone diseases and for assessing the P intake in pigs (Boyd et al. 1983). The aim of the present study was to screen the polymorphisms in selected genes involved in mineral and bone metabolism and to examine their association with the serum levels of P, Ca, ALP, and the Ca/P ratio. Consequently, the results will contribute to unravel the genetic background of the phenotypical variation of P utilization and enable the selection for improved P efficiency in pig breeding programs.

## Material and methods

### Animals, sample collection, and use

Animal care and sample collection procedures followed the guidelines of the German Law of Animal Protection. The experimental protocol was approved by the Animal Care Committee of the Leibniz Institute for Farm Animal Biology (FBN). All relevant international, national, and/or institutional guidelines for the care and use of animals have been met. The study comprised a population of German Landrace pigs (DL; *n* = 360). Groups of ten animals were housed in fully slatted pens. Pigs had ad libitum access to standard feed and water. The tested animals were sired by 18 boars and weighed between 92 and 132 kg. The herd comprised 90 females, 76 intact males, and 187 castrated males. Pigs were killed by electrical stunning followed by exsanguination in the experimental slaughterhouse of FBN. Trunk blood was collected and serum samples were prepared by centrifugation of clotted blood for 20 min at 4 °C and 3500×*g*. Serum samples were stored at − 80 °C until use for measurement of serum parameters (inorganic phosphate, calcium, and alkaline phosphatase) with commercial assays using Fuji DriChem 4000i (FujiFilm, Minato, Japan). Furthermore, liver tissue was collected and genomic DNA was isolated. Therefore, samples were lysed with proteinase K (Roth, Karlsruhe, Germany) followed by a phenol-chloroform extraction using Phase Lock Gel tubes (5 Prime, Hamburg, Germany) according to the manufacture’s recommendations. The DNA was precipitated, resuspended in Tris-EDTA buffer, and stored at − 20 °C until use.

### Single nucleotide polymorphism detection

For the in silico detection of DNA polymorphisms, sequence data of candidate genes were retrieved from the Ensembl genome browser, based on *Sus scrofa* genome build 10.2 (Ensembl 89; accessed on July 2017). The sequence data was used to select regions with interesting SNPs, preferentially located in exons and showing multiple observations known as “evidence status” (Ensembl database). Selected SNPs (Table 1) were screened for segregation by comparative sequencing of PCR fragments in pooled samples of animals with either low or high serum P levels. Polymerase chain reactions (PCR) were performed in a 20-μl volume containing 100 ng of genomic DNA, 1× PCR buffer (with 1.5 mM MgCl_2_), 0.25 mM of dNTP, 0.2 μM of each primer, and 0.5 U of Taq DNA polymerase (GeneCraft, Münster, Germany). The PCR procedures were performed via initial denaturing at 94 °C for 4 min followed by 40 cycles of 30 s at 94 °C, 30 s at 60 °C, 1 min at 72 °C, and final elongation of 5 min at 72 °C. The PCR products were checked for specific amplification on 1.5% agarose gels and purified with Agencourt AMPure XP (Beckman Coulter, Krefeld, Germany) and sequenced in either forward or reverse direction using an ABI3130 DNA Analyzer. The details of genes and corresponding primer sequences are listed in Supplementary Table S1.

**Table 1:** Methods used for SNP detection and genotyping in porcine genes relevant in bone and mineral metabolism

Gene	Reference SNP ID	Origin/source	Genotyping method
CALCR	rs81218770	In-house database, validated by KASP	KASP
CASR	rs81448439	Pool sequencing	KASP
FGF23	rs710498025	Pool sequencing	KASP
PTH1R	rs330276009	Rangkasenee et al. (2013)	RFLP
SPP1	rs81214161	Pool sequencing	KASP
STC1	rs80787827	Pool sequencing	RFLP
TRAFD1	rs345195312	Pool sequencing	RFLP
VDR	rs323540588	Pool sequencing	KASP

### Genotyping

For genotyping by restriction fragment length polymorphism (RFLP), the amplification of genomic target sequences was performed in a standard PCR using gene specific primers as described above. The PCR products were digested by the restriction enzyme *MboII*, *HphI*, and *XhoI* (New England BioLabs, Frankfurt am Main, Germany) for *PTH1R*, *TRAFD1*, and *STC1*, respectively. Digestion of amplification products was performed using 10 μl PCR product, 2 μl CutSmart Buffer (New England BioLabs), and 1 U of restriction enzyme and filled up with *aqua dest* to a final volume of 20 μl. The reaction conditions were 16 h (for *PTH1R* and *TRAFD1*) or 2 h (for *STC1*) at 37 °C for incubation and 20 min at 65 °C for enzyme inactivation. The resulting fragment lengths are listed in Supplementary Table S1. The digested products were separated and analyzed on 3% agarose gels.

The KASP genotyping assays for SNPs in *CALCR*, *FGF23*, *VDR*, *CASR*, and *SPP1* were designed by LGC genomics (Hoddesdon, UK; Supplementary Table S1). The KASP assays were performed in a 10-μl volume containing 100 ng of genomic DNA, 5 μl of KASP Master mix, and 0.14 μl of SNP-specific KASP Assay mix. The touchdown PCR program had following conditions: initial activation at 94 °C for 15 min followed by 10 cycles of 20 s at 94 °C for denaturation, 60 s at 61–55 °C (drop of 0.6 °C per cycle) for annealing and elongation, followed by 26 cycles of 20 s at 94 °C and 60 s at 55 °C. The assay results were analyzed at 37 °C using a LightCycler®480 system (Roche, Mannheim, Germany).

### Statistical analysis

The distribution of measured serum parameters within the analyzed pig population was visualized using R version 3.3.1. A mixed linear model (JMP Genomics 7.0, SAS Institute, Cary, NC, USA) including sex as fixed effect and sire as random effect was applied to estimate impact of sex on analyzed serum parameters (Supplementary Table S1). The association between SNPs and serum parameters was analyzed using a mixed linear model (JMP Genomics 7.0, SAS Institute, Cary, NC, USA). The model included SNP genotype and sex as fixed effect, sire as random effect, and slaughter weight as a covariate. Least square means for genotypes were compared by Tukey’s range test. Results were considered significant at *p* ≤ 0.05. A tendency was considered at *p* ≤ 0.10. In order to account for type I errors, multiple-testing correction was performed with JMP Genomics 7.0, whereby the false discovery rate (*q* value) was calculated (Storey and Tibshirani 2003). Allele and genotype frequencies were calculated and tested for Hardy-Weinberg equilibrium (HWE) by chi-square analysis using the package *genetics* in R. Significant deviation from HWE was assumed at *p* ≤ 0.05.

## Results

### Serum parameters and single nucleotide polymorphisms

**Fig. 2:**
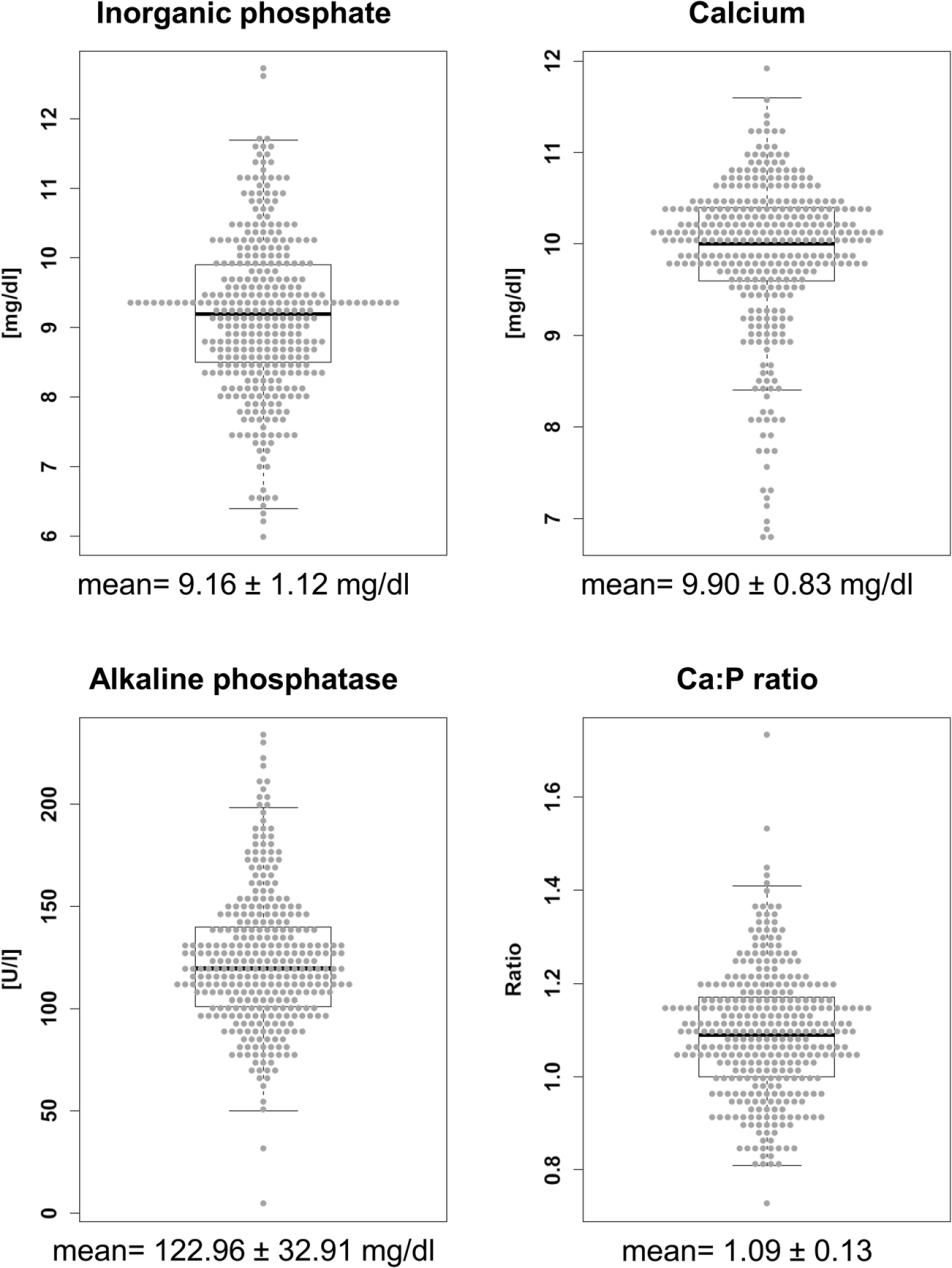
Boxplots displaying the phenotypical variation of phosphorus relevant serum parameters in the tested German Landrace population

Phosphorus-related serum parameters showed a phenotypical variation in the tested German Landrace population (Fig. 2). The inorganic phosphate levels ranged between 6.0 and 12.7 mg/dl and the calcium levels between 6.8 and 11.9 mg/dl. The alkaline phosphatase exhibited the broadest variance (SD = 32.91 U/l) and ranged between 5 and 233 U/l. The Ca/P ratios varied between 0.73 and 1.73. Allele and genotype frequencies of selected SNPs located in candidate genes are listed in Table 2. Moreover, sequencing of pooled samples revealed additional variants reported in Supplementary Table S1. The selected SNPs of *CALCR* and *STC1* are located in the 3′UTR; the *VDR* SNP is located in a non-coding region. The remaining SNPs are located in coding regions, being either silent (*CASR*, *SPP1* and *TRAFD1*) or causing an amino acid exchange (*FGF23* and *PTH1R*). The SNP in *PTH1R* was described previously by Rangkasenee et al. (2013). The chi-square analysis revealed that the genotype distribution of *CALCR* departures from HWE, whereas the other loci are in HWE.

**Table 2:** Allele and genotype frequencies in genes relevant in bone and mineral metabolism in the tested German Landrace population

Gene (SNP ID)	Position	Allele	Allele frequency	Genotype	Number of animals (genotype frequency)	HWE^c^
CALCR (rs81218770)	3′UTR	C	0.17	CC	4 (0.01)	*p* < 0.05
		G	0.83	CG	116 (0.32)	
				GG	239 (0.67)	
CASR (rs81448439)	Coding	A	0.46	AA	76 (0.20)	NS
	(synonymous)	G	0.54	AG	194 (0.51)	
				GG	108 (0.29)	
FGF23 (rs710498025)	Coding	A	0.86	AA	266 (0.74)	NS
	(p.Arg94Gly)^a^	G	0.14	AG	82 (0.23)	
				GG	11 (0.03)	
PTH1R (rs330276009)	Coding	C	0.77	CC	203 (0.60)	NS
	(p.Leu556Phe)^b^	T	0.23	CT	120 (0.35)	
				TT	18 (0.05)	
SPP1 (rs81214161)	Coding	A	0.17	AA	15 (0.04)	NS
	(synonymous)	C	0.83	AC	91 (0.25)	
				CC	254 (0.71)	
STC1 (rs80787827)	3′UTR	A	0.85	AA	247 (0.71)	NS
		G	0.15	AG	96 (0.28)	
				GG	5 (0.01)	
TRAFD1 (rs345195312)	Coding	A	0.55	AA	102 (0.29)	NS
	(synonymous)	G	0.45	AG	188 (0.53)	
				GG	66 (0.19)	
VDR (rs323540588)	Intron	C	0.73	CC	200 (0.53)	NS
		T	0.27	CT	149 (0.40)	
				TT	26 (0.07)	

^a^Reference sequence: ENSSSCP00000024420

^b^Reference sequence: ENSSSCP00000012077

^c^HWE: test for deviation from Hardy-Weinberg equilibrium by chi-square analysis (NS—no significant deviation; *p* < 0.05—significant deviation)

### Association analysis

The association analysis between the SNPs and serum parameters are summarized in Table 3. The statistical analysis revealed that the analyzed SNP in the *FGF23* locus was associated with the Ca/P ratio. In addition, the comparison of certain genotype classes for *CASR*, *STC1*, and *TRAFD1* were assessed as significantly different (*p* ≤ 0.05). Specifically, the homozygous GG genotype of *CASR* rs81448439 and the homozygous AA genotype of *STC1* rs80787827 were associated with higher Ca/P ratios. Animals with the heterozygous AG genotype of *FGF23* (rs710498025) showed higher Ca/P ratios compared to carriers of the alternative homozygous allele. The homozygous GG genotype of the analyzed SNP rs345195312 in *TRAFD1* was associated with a reduced Ca/P ratio. Furthermore, the *TRAFD1* SNP was significantly (*p* ≤ 0.05) associated with inorganic P levels, where animals carrying the GG genotype exhibited higher serum P levels. *CALCR*, *PTH1R*, *SPP1*, and *VDR* polymorphisms lack any associations with the serum parameters. Moreover, due to multiple testing (false discovery rate), none of the revealed associations were significant (*q* value > 0.05). Serum ALP was not significantly associated with the respective SNPs, but with sex of the animals (Supplementary Table S1). Castrated males revealed the highest serum ALP levels compared to females and intact males. Slaughter weight was significantly associated (*p* ≤ 0.05) with serum P concentrations and Ca/P ratio in all conducted analyses.

**Table 3:** Association of candidate gene SNPs with serum parameters of P metabolism in the tested German Landrace population

Gene	Trait	Genotype LSM^a^	SE	Genotype LSM	SE	Genotype LSM	SE	*p* value	*q* value
CALCR		CC		CG		GG			
	Inorganic phosphate (mg/dl)	8.59	0.63	9.11	0.12	9.08	0.10	0.704	0.967
	Calcium (mg/dl)	9.62	0.49	9.83	0.09	9.89	0.06	0.722	0.967
	Ca/P ratio	1.13	0.08	1.09	0.01	1.11	0.01	0.405	0.800
	Alkaline phosphatase (U/l)	96.30	17.83	117.49	4.40	122.95	4.13	0.157	0.615
CASR		AA		AG		GG			
	Inorganic phosphate (mg/dl)	9.22	0.15	9.15	0.10	8.94	0.13	0.276	0.736
	Calcium (mg/dl)	9.79	0.10	9.85	0.07	9.96	0.09	0.467	0.800
	Ca/P ratio	1.08a	0.02	1.09ab	0.01	1.13b	0.01	0.065	0.608
	Alkaline phosphatase (U/l)	119.43	5.02	121.46	3.94	115.68	4.58	0.395	0.800
FGF23		AA		AG		GG			
	Inorganic phosphate (mg/dl)	9.11	0.09	8.89	0.14	9.47	0.34	0.136	0.615
	Calcium (mg/dl)	9.87	0.06	9.93	0.10	9.49	0.25	0.274	0.736
	Ca/P ratio	1.10ab	0.01	1.13a	0.02	1.02b	0.04	*0.020*	0.496
	Alkaline phosphatase (U/l)	121.87	4.08	115.24	5.15	119.85	10.71	0.359	0.800
PTH1R		CC		CT		TT			
	Inorganic phosphate (mg/dl)	9.12	0.11	9.06	0.12	9.04	0.28	0.881	0.967
	Calcium (mg/dl)	9.86	0.06	9.91	0.08	9.85	0.21	0.861	0.967
	Ca/P ratio	1.10	0.01	1.11	0.01	1.10	0.03	0.824	0.967
	Alkaline phosphatase (U/l)	120.26	4.18	121.71	4.48	120.93	8.99	0.937	0.967
SPP1		AA		AC		CC			
	Inorganic phosphate (mg/dl)	8.98	0.33	9.18	0.15	9.07	0.11	0.655	0.967
	Calcium (mg/dl)	9.97	0.23	10.02	0.09	9.81	0.06	0.152	0.615
	Ca/P ratio	1.11	0.04	1.10	0.02	1.10	0.01	0.922	0.967
	Alkaline phosphatase (U/l)	121.73	10.06	120.13	5.01	120.56	4.03	0.983	0.983
STC1		AA		AG		GG			
	Inorganic phosphate (mg/dl)	8.22	0.54	9.09	0.14	9.12	0.10	0.256	0.736
	Calcium (mg/dl)	10.04	0.43	9.91	0.10	9.85	0.06	0.825	0.967
	Ca/P ratio	1.23a	0.07	1.10ab	0.02	1.09b	0.01	0.110	0.615
	Alkaline phosphatase (U/l)	118.04	15.46	123.49	4.81	119.01	3.92	0.573	0.917
TRAFD1		AA		AG		GG			
	Inorganic phosphate (mg/dl)	8.98a	0.12	9.04a	0.09	9.43b	0.15	*0.031*	0.496
	Calcium (mg/dl)	9.82	0.09	9.90	0.07	9.90	0.12	0.750	0.967
	Ca/P ratio	1.11ab	0.01	1.11a	0.01	1.06b	0.02	0.076	0.608
	Alkaline phosphatase (U/l)	120.24	4.86	122.10	4.23	115.66	5.60	0.427	0.767
VDR		CC		CT		TT			
	Inorganic phosphate (mg/dl)	9.17	0.11	8.93	0.12	9.09	0.24	0.173	0.615
	Calcium (mg/dl)	9.89	0.07	9.80	0.08	10.01	0.18	0.473	0.800
	Ca/P ratio	1.09	0.01	1.11	0.01	1.12	0.03	0.456	0.800
	Alkaline phosphatase (U/l)	119.97	4.23	121.15	4.50	118.78	7.57	0.922	0.967

*LSM* least square mean, *SE* standard error

^a^LSM with different lowercase letters (a, b) differ at *p* < 0.05

## Discussion

The use of serum parameters reflect the ingested amount of minerals and provide diagnostic value for bone diseases as described previously (Boyd et al. 1983). Secondly, blood sample collection is minimally invasive. Recently, high heritability estimates for serum P, Ca, and ALP concentrations were revealed by microsatellite analysis of an F2 intercross between Landrace and Korean native pigs (Yoo et al. 2012), indicating a prominent role of genetics in the variability of these parameters. There are a few studies addressing the genetic background of serum parameter in pigs, particularly with regard to the health status of animals (Reiner et al. 2008; Yoo et al. 2012; Bovo et al. 2016).

In this study, eight candidate genes involved in mineral and bone metabolism were selected to perform an association study with serum levels of P, Ca, ALP, and the Ca/P ratio. Significant associations at a nominal *p* value of *p* ≤ 0.05 were uniquely found for SNPs located in *FGF23* and *TRAFD1*.

Animals with the heterozygous AG genotype of *FGF23* rs710498025 showed higher Ca/P ratios. This SNP is a missense variant, causing an amino acid exchange from arginine to glycine (p.Arg94Gly) in the conserved FGF family domain. The effect on Ca/P ratio may base on the phosphaturic effect mediated by FGF23 hormone, which enhance the renal P excretion and P fluxes from bone. In human, *FGF23* was reported to be associated with serum P concentration (Kestenbaum et al. 2010). As the association analysis lacks significance after multiple testing, it might be relevant to either test other variants in the *FGF23* locus or reproduce the results in independent populations or other breeds. However, despite the analyzed SNP rs710498025, no additional sequence variations were identified in the coding region of FGF23 in the investigated German Landrace pigs.

The homozygous GG genotype of the *TRAFD1* SNP rs345195312 was significantly (*p* ≤ 0.05) associated with higher serum P concentrations. Interestingly, the gene is located in a suggestive quantitative trait locus (QTL) for serum Ca levels in Landrace × Korean native pig resource population (Yoo et al. 2012). Moreover, the *TRAFD1* gene is also located in a QTL for osteochondrosis score in a Duroc × Pietrain resource population (Laenoi et al. 2011), indicating an involvement in bone metabolism. Indeed, *TRAFD1* mediates the resorbing and secretory activity of osteoclasts (Witwicka et al. 2015). Furthermore, *TRAFD1* is suggested to induce inflammatory expression of *MMP13* in osteoarthritis (Radwan et al. 2015). The nominal significant association with serum P levels and literature evidence for its contribution to bone disorders emphasize *TRAFD1* as a promising candidate gene for further analyses regarding the connection between P homeostasis and immune system.

In addition, distinct genotype contrasts of the analyzed *CASR*, *STC1* and *TRAFD1* SNPs revealed nominal significant associations. Animals with the homozygous GG genotype of *CASR* rs81448439 have a higher Ca/P ratio compared to those having AA genotype (*p* ≤ 0.05). Since the Ca/P ratio is independent of feed intake, this might indicate an effect on the homeostatic balance between Ca and P. Previous studies demonstrated that higher Ca/P ratios reduce the P absorption, while Ca absorption is increased (Koch and Mahan 1986). Although this SNP is a synonymous variant, eight other variants including one splice region variant were detected for the sequenced *CASR* fragment in the German Landrace population and warrants further investigation (Supplementary Table S1). Bovo et al. (2016) rejected *CASR* as a candidate gene for affecting serum Ca in pigs, based on their results deduced from an Italian Large White population. Nevertheless, *CASR* is not only responsible for Ca homeostasis but is also involved in bone metabolism, such as the proliferation of bone marrow mesenchymal stem cells (Ye et al. 2016).

Regarding the *STC1* SNP rs80787827, carriers of the AA genotype showed an increased Ca/P ratio compared to pigs exhibiting the GG genotype. Due to increasing serum P levels and decreasing serum Ca levels, the G allele might be favorable with regard to hydroxyapatite deposition in bone (Madsen et al. 1998). The analyzed SNP is located in the 3′UTR, which seems to be well conserved between mammalian species (Varghese et al. 1998) and which was suggested to be important to understand the molecular mechanisms of gene regulation (Hung et al. 2012). Furthermore, the involvement of *STC1* in bone formation is supported by its location in a QTL for osteochondrosis-related traits in different pig populations (Lee et al. 2003; Laenoi et al. 2011*)*.

In this study, a significant association of sequence variants of *CALCR*, *PTH1R*, *SPP1*, and *VDR* with serum parameters was not observed, even though *CALCR* has been associated with leg locomotion traits (Fan et al. 2009) and bone integrity (Alexander et al. 2010), and *PTH1R* has been associated with osteochondrosis lesions in pigs (Rangkasenee et al. 2013). Although, in human, there are some polymorphisms of *SPP1* known to be associated with the formation of urinary Ca stones (Gao et al. 2005), no significant associations were revealed between *SPP1* SNP rs81214161 and serum parameters. However, calcifications of the urinary tract might be not reflected by the analyzed serum traits in pigs. The *VDR* gene, which not only plays a central role in the regulation of the P and Ca homeostasis but also influences the expression of various other target genes, might play a greater role in the variation of serum traits than the association analysis of the identified intron variant rs323540588 has shown. In human, it is known that mutations in the VDR sequence could have an effect on RXR heterodimerization, thus modulating the VDRE binding to target genes (Haussler et al. 2011).

It should be noted that the analyzed *CALCR* SNP rs81218770 revealed a significant deviation from HWE. However, commercial pigs were under artificial selection, which affected allele frequencies and the extent of linkage disequilibrium (Amaral et al. 2008). Furthermore, the results from Fan et al. (2009) and Alexander et al. (2010) based on an intron variant (rs81218964) of CALCR analyzed in other pig breeds. This intron variant deviates also from HWE in a Berkshire × Yorkshire (genotype count: GG-494; AG-77; AA-1) and Large White × Landrace (genotype count: GG-1172; AG-764; AA-115) population (Ensembl database, accessed on August 30, 2017), supporting that the *CALCR* loci is under selection in domesticated pigs.

In this study, none of the analyzed polymorphisms was associated with serum ALP. Two studies detected a putative QTL for serum ALP on pig chromosome SSC6 for a Meishan × Pietrain cross and a Landrace x Korean native pig population (Reiner et al. 2008; Yoo et al. 2012). Reiner et al. (2008) proposed the tissue non-specific *ALPL* gene as a positional candidate because of the co-localization to the QTL on SSC6. Thus, the ALPL gene could be a promising candidate for serum ALP in German Landrace pigs in further studies.

The fact that all associations do not differ significantly after correction for multiple testing may indicate that the genetics of the major regulators of P and Ca homeostasis focused in this study make only a small contribution to the considerable variability of the serum traits. This corresponds in particular for phosphorus to the results of genome-wide association analyses, which also pointed out genes with yet unknown function in P-homeostasis (Bovo et al. 2016). With regard to the observed nominally significant associations, the corresponding linkage block could be further investigated in order to uncover causal source for trait variations. In general, further polymorphisms in other regions of these genes might, however, be considered to obtain a broader picture of their involvement in the complex traits studied.

In summary, the results promote *FGF23* and *TRAFD1* as most promising candidate genes for mineral utilization and homeostasis in pigs. In contrast, variants in genes such as *VDR*, *SPP1*, *CALCR*, and *PTH1R*, being considered as major regulators of P and Ca homeostasis, were not found to be significantly associated with the analyzed traits in the tested pig population. Hence, the phenotypical variation of serum parameters (Fig. 2) might rely on genomic regions and gene variants to be elucidated via holistic approaches such as genome-wide association study (GWAS).

## Electronic supplementary material

ESM 1 (XLSX 22 kb)
